# Colonic Leiomyosarcoma Treated With Laparoscopy-Endoscopy Cooperative Surgery for Colorectal Tumors (LECS-CR) Resulting in Peritoneal Dissemination Recurrence: A Case Report

**DOI:** 10.7759/cureus.107377

**Published:** 2026-04-20

**Authors:** Shunta Ichikawa, Michitoshi Takano, Hirosuke Inoue, Tomomasa Fukasawa, Hisato Higashi

**Affiliations:** 1 Surgery, Japan Community Healthcare Organization Tokyo Shinjuku Medical Center, Tokyo, JPN

**Keywords:** colon, laparoscopy-endoscopy cooperative surgery, lecs-cr, leiomyosarcoma, recurrence, submucosal tumor

## Abstract

Gastrointestinal leiomyosarcoma is a rare tumor, particularly in the colon. We report a case of colonic leiomyosarcoma treated with laparoscopy-endoscopy cooperative surgery for colorectal tumors (LECS-CR) and discuss its feasibility and limitations. A 57-year-old man presented with a submucosal tumor in the ascending colon detected following a positive fecal occult blood test. LECS-CR was performed for both diagnostic and therapeutic purposes. Histopathological examination confirmed leiomyosarcoma. Although complete resection was achieved, peritoneal dissemination recurrence at the anastomotic site was observed one year postoperatively, requiring open right hemicolectomy. No recurrence has been observed during the six months following the second surgery. While LECS-CR may represent a minimally invasive treatment option, strategies to prevent tumor dissemination associated with bowel opening procedures remain a critical issue. This case highlights the potential limitations of LECS-CR in leiomyosarcoma and underscores the importance of appropriate patient selection and postoperative surveillance strategies.

## Introduction

Colonic leiomyosarcoma is an extremely rare malignant tumor, accounting for a small proportion of gastrointestinal mesenchymal neoplasms. Following the discovery of c-kit and the establishment of gastrointestinal stromal tumor (GIST) as a distinct entity, many tumors previously diagnosed as leiomyosarcoma have been reclassified as GIST, further highlighting the rarity of true leiomyosarcoma of the gastrointestinal tract [1,2]. Leiomyosarcoma can occur throughout the gastrointestinal tract, including the colon and rectum, although its distribution varies among reports.

Unlike colorectal adenocarcinoma, lymph node metastasis is considered relatively uncommon in gastrointestinal leiomyosarcoma [3]. Consequently, the optimal surgical strategy, including the extent of resection, has not been established. Recently, minimally invasive approaches such as laparoscopy-endoscopy cooperative surgery for colorectal tumors (LECS-CR) have been developed and applied to selected colorectal lesions, particularly submucosal tumors. However, concerns remain regarding oncological safety, including the potential risk of tumor dissemination associated with bowel opening procedures [4,5]. In this context, given the rarity of colonic leiomyosarcoma and the limited evidence regarding the application of LECS-CR for this tumor, this case provides important insights into its feasibility and limitations, particularly in terms of oncological safety and postoperative management.

## Case presentation

A 57-year-old man presented in August 2023 with a positive fecal occult blood test, without specific gastrointestinal symptoms. His medical history included dyslipidemia, and he was taking rosuvastatin calcium. Physical examination revealed no remarkable findings, and no palpable abdominal mass was detected.

**Figure 1:**
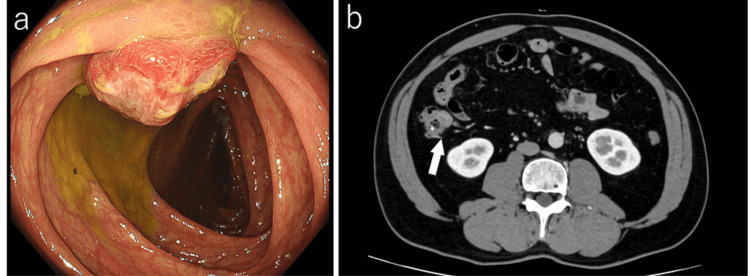
Colonoscopy and contrast-enhanced abdominal CT findings (a) Colonoscopy revealed a 20 mm elevated lesion with central ulceration in the ascending colon. (b) On contrast-enhanced abdominal CT, the clip placed near the tumor site was identified; however, the tumor itself was not clearly visualized. No liver metastases or ascites were observed.

Colonoscopy revealed a 20 mm elevated lesion with ulceration in the ascending colon (Figure 1a). Histopathological examination of biopsy specimens suggested a smooth muscle tumor.

Contrast-enhanced CT demonstrated a clip placed near the lesion, which had been endoscopically placed during colonoscopy to mark the tumor site; however, the tumor itself was not clearly visualized, and no liver metastases or ascites were observed (Figure 1b). Histopathological examination revealed fascicular proliferation of spindle-shaped cells with enlarged oval to spindle-shaped nuclei. Immunohistochemically, DOG-1 and c-kit were negative, excluding GIST. In contrast, smooth muscle actin (SMA) and desmin were positive, suggesting a smooth muscle tumor. In addition, CD34 and S-100 were also negative.

Because the lesion was a submucosal tumor, endoscopic submucosal dissection was not indicated. Although biopsy could not determine malignancy, even in the case of leiomyosarcoma, the significance of lymph node dissection remains unclear. Based on these findings, given the submucosal nature of the lesion and the uncertainty regarding malignancy, standard right hemicolectomy was considered potentially overly invasive.

Therefore, LECS-CR was selected for both diagnostic and therapeutic purposes. The treatment strategy was determined after discussion with endoscopy specialists experienced in LECS-CR, based on the clinical, endoscopic, and pathological findings.

Surgery was performed under general anesthesia in the lithotomy position. Four ports were placed: a 12 mm camera port at the umbilicus, a 12 mm port in the left upper abdomen, a 5 mm port in the left lower abdomen, and a 5 mm port in the upper midline. Endoscopy revealed a tumor at the hepatic flexure of the ascending colon. After submucosal injection of glycerol, a circumferential mucosal incision was made using a needle knife.

Based on the endoscopic circumferential marking, laparoscopy confirmed adequate margins, and full-thickness resection was initiated using an electrosurgical knife, with intentional perforation to complete approximately half of the circumference. After confirming adequate margins laparoscopically, full-thickness resection was completed, and the remaining half of the bowel wall was divided using an ultrasonic coagulating device. The specimen was retrieved without the use of a specimen retrieval bag.

To prevent leakage of intestinal contents into the abdominal cavity, temporary closure was performed with three stitches of 3-0 absorbable monofilament sutures, which also served as traction sutures. Indocyanine green was administered intravenously to confirm adequate blood flow, and the defect was closed using two linear staplers. Blood loss was minimal, and the operative time was two hours and 33 minutes.

**Figure 2:**
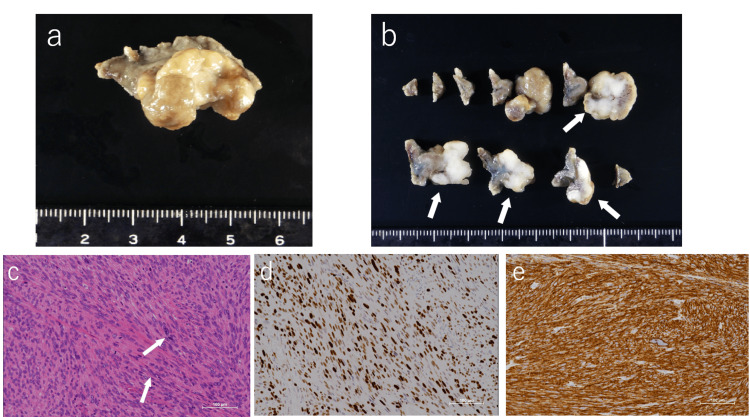
Macroscopic, histopathological, and immunohistochemical findings (a, b) Gross pathological findings of the resected specimen revealed a 33 × 15 mm sample containing a protruding lesion measuring 23 × 16 × 18 mm. On the cut surface, a solid, whitish tumor (white arrows) was observed. (c) Histopathological findings revealed fascicular proliferation of spindle-shaped cells on hematoxylin and eosin staining, similar to the endoscopic biopsy. Focal nuclear enlargement and occasional mitotic figures (white arrows) were observed (200x). (d) The Ki-67 proliferative index (assessed with MIB-1 antibody) was estimated to be 60%-70% (×200). (e) Caldesmon, a smooth muscle-specific marker, was positive on immunohistochemistry.

Gross examination revealed a specimen measuring 33 × 15 mm containing a protruding lesion measuring 23 × 16 × 18 mm. The cut surface showed a solid, whitish tumor (Figures 2a, 2b).

Histopathological examination revealed fascicular proliferation of spindle-shaped cells with nuclear atypia and mitotic figures (Figure 2c). Ki-67 proliferative index (assessed with MIB-1 antibody) was 60%-70% (Figure 2d). Immunohistochemistry showed DOG-1(-), CD34(-), desmin(+), and caldesmon(+), leading to a diagnosis of leiomyosarcoma (Figure 2e).

The resection margin was histologically negative, with a minimum margin distance of 6 mm; however, the tumor was located close to the submucosal layer. Intraoperative frozen section analysis was not performed.

The patient was followed according to colorectal cancer surveillance protocols, with blood tests every three months and a CT every six months. One year postoperatively, CT revealed a mass suspicious for peritoneal dissemination around the anastomosis. Fluorodeoxyglucose (FDG)-PET subsequently demonstrated uptake corresponding to the mass at the anastomotic site, with a maximum standardized uptake value (SUVmax) of 4.7. Although the SUV value was relatively low, malignancy was suspected based on the concordance with the CT findings.

Open right hemicolectomy was performed for recurrence. Intraoperatively, a tumor was palpated at the anastomosis, and three nodules (two in the greater omentum and one in the small bowel mesentery) were resected. Histopathological examination confirmed recurrent leiomyosarcoma with peritoneal dissemination, and the patient remains recurrence-free six months after the second surgery.

## Discussion

Following the discovery of c-kit and the establishment of the concept of GIST, many tumors previously diagnosed as leiomyosarcoma have been reclassified as GIST. Therefore, true leiomyosarcoma is now considered rare. Leiomyosarcoma can occur throughout the gastrointestinal tract, including the stomach, small intestine, and colon [3].

The five-year survival rate of gastrointestinal leiomyosarcoma has been reported to be 51.6% [3]. Poor prognostic factors include distant metastasis, a mitotic count of ≥5 per 10 high-power fields (HPF), colonic origin, and a Ki-67 labeling index ≥ 10% [6]. Complete surgical resection is the standard treatment for colonic leiomyosarcoma. Hematogenous metastasis and peritoneal dissemination are considered the main routes of spread; however, lymph node metastasis has been reported in approximately 10% of cases [7]. The clinical significance of lymph node dissection remains unclear.

A literature search of Ichushi-Web was performed for cases reported after the establishment of the diagnostic concept of GIST in the early 2000s, using the keywords “colonic leiomyosarcoma,” “colon leiomyosarcoma,” and “rectal leiomyosarcoma.” This search identified 20 reported cases (excluding conference abstracts), which are summarized in Table 1. Regarding lymph node dissection, D3 dissection was performed in eight cases, D2 in three cases, and D1 in one case, while the extent of dissection was not described in eight cases. All patients underwent standard colectomy for colorectal cancer, and no cases of local resection were reported. No lymph node metastasis was observed at diagnosis or during follow-up, suggesting that extensive lymphadenectomy, similar to that performed for colorectal cancer, may be overly invasive.

**Table 1:** Comparison between our case and previously reported cases C: cecum; A: ascending colon; T: transverse colon; D: descending colon; S: sigmoid colon; R: rectum; APR: abdominoperineal resection; LAR: low anterior resection; SC: sigmoid colectomy; TC: transverse colectomy; ICR: ileocecal resection; RHC: right hemicolectomy; LHC: left hemicolectomy; ND: not described; LECS-CR: laparoscopy-endoscopy cooperative surgery for colorectal tumors

Case No.	Author	Year	Age	Sex	Location	Tumor size (mm)	Treatment	Lymphadenectomy	Metastasis
1	Tokuyama et al. [8]	2004	81	M	S	130	SC	ND	Peritoneum (metachronous)
2	Shinofuji et al. [9]	2004	58	M	R	85	APR	D2	Liver (metachronous)
3	Kato et al. [10]	2008	59	M	C	95	RHC with partial hepatectomy	D3	Liver (metachronous)
4	Myoga et al. [11]	2009	63	F	T	50	TC	D1	None
5	Oishi et al. [12]	2011	53	M	A	50	ICR	ND	Liver (synchronous)
6	Higashijima et al. [13]	2012	60	M	S	40	SC	D3	None
7	Fukada et al. [14]	2013	53	M	T	35	RHC	D3	None
8	Kimura et al. [15]	2013	72	M	R	23	APR	ND	None
9	Ogiku et al. [16]	2015	61	M	R	ND	LAR	ND	Liver, lung (metachronous)
10	Kono et al. [17]	2015	46	M	T	120	RHC	ND	Peritoneum, liver (metachronous)
11	Ito et al. [18]	2015	72	M	A	130	ICR	ND	None
12	Osaragi et al. [19]	2016	88	F	A	40	RHC	D2	Lung (metachronous)
13	Akutsu et al. [20]	2016	51	F	D	40	Partial resection of the D	ND	None
14	Ikeshoji et al. [21]	2017	50	F	A	65	RHC	D2	None
15	Hoshino et al. [22]	2017	87	M	R	7.5	APR	ND	Lung (metachronous)
16	Fujita et al. [23]	2018	69	M	D	ND	LHC	D3	None
17	Yoshida et al. [24]	2018	78	M	A	100	RHC	D3	Liver (metachronous)
18	Manabe et al. [25]	2018	41	F	A	100	TC	D3	None
19	Kobayashi et al. [26]	2020	88	M	A	250	RHC	D3	None
20	Miyamoto et al. [27]	2022	61	F	T	50	TC	D3	Liver (metachronous)
21	Our case		57	M	A	20	LECS-CR	None	Peritoneum (metachronous)

A further literature search was performed using Ichushi-Web and PubMed with the keywords “colonic leiomyosarcoma,” “LECS,” and “laparoscopy-endoscopy cooperative surgery.” No clearly relevant reports were identified in these databases. To the best of our knowledge, such cases are rare. In the present case, LECS-CR enabled local resection and may contribute to expanding treatment options; however, recurrence at the anastomotic site indicates that careful consideration of oncological safety is required.

In the present case, endoscopic ultrasound (EUS) was not performed because the lesion was located in the ascending colon, where the application of EUS is technically limited. The clip identified on CT had been placed endoscopically during colonoscopy to mark the lesion site. Although the tumor measured approximately 20 mm, it was not clearly visualized on contrast-enhanced CT. This may be explained by its intramural location, attenuation similar to the surrounding bowel wall, and limited vascular enhancement.

According to Sakamoto et al. [4], the indications for LECS-CR include (1) recurrent intramucosal cancer after endoscopic or local surgical treatment with severe fibrosis; (2) intramucosal cancer extending into adenomas, diverticula, or the appendix; and (3) adenomas and submucosal tumors (e.g., GIST, leiomyoma, and schwannoma). However, concerns have been raised regarding tumor cell dissemination during bowel opening. In the present case, although surgical margins were negative, the high MIB-1 index (60%-70%) suggests a high risk of recurrence. Additionally, ulceration observed endoscopically suggests possible mucosal invasion, which may have contributed to tumor cell dissemination during bowel opening.

Future challenges include improving surgical techniques to prevent tumor dissemination, such as non-exposure techniques and the use of closure devices [5]. Intraoperative frozen section analysis may also be useful for ensuring adequate margins. To our knowledge, such cases are rare, and further accumulation of cases and long-term follow-up are required. In particular, tumor proliferative activity, margin management, and prevention of peritoneal dissemination should be investigated in future multicenter studies. In particular, tumor proliferative activity, margin management, and prevention of peritoneal dissemination should be investigated in future multicenter studies.

This study has several limitations. First, mitotic count and necrosis were not quantitatively assessed. Second, additional immunohistochemical markers such as STAT6 and ALK were not evaluated. Third, intraoperative frozen section analysis was not performed. Fourth, a specimen retrieval bag was not used during specimen extraction. Finally, postoperative surveillance was performed based on colorectal cancer protocols, which may not be appropriate for leiomyosarcoma, which predominantly recurs via hematogenous spread. Surveillance strategies based on soft tissue sarcoma guidelines, including more frequent imaging and chest CT, may have been more appropriate, particularly for high-grade tumors. These limitations should be considered when interpreting the findings of this case.

## Conclusions

We report a case of colonic leiomyosarcoma treated with LECS-CR. Although LECS-CR may represent a minimally invasive treatment option, its application to leiomyosarcoma should be carefully considered, as it may fall outside the conventional indications for this approach. In the present case, recurrence occurred after surgery, and both hematogenous spread and intraoperative tumor dissemination are possible mechanisms; therefore, causality cannot be established from a single case. In addition, postoperative surveillance based on colorectal adenocarcinoma protocols may have been suboptimal. Careful patient selection, appropriate surveillance strategies, and further accumulation of cases with long-term follow-up are required to clarify its oncological safety.
